# Systematic Identification of Spontaneous Preterm Birth-Associated RNA Transcripts in Maternal Plasma

**DOI:** 10.1371/journal.pone.0034328

**Published:** 2012-04-05

**Authors:** Stephen S. C. Chim, Wing S. Lee, Yuen H. Ting, Oi K. Chan, Shara W. Y. Lee, Tak Y. Leung

**Affiliations:** 1 Department of Obstetrics and Gynaecology, The Chinese University of Hong Kong, Shatin, New Territories, Hong Kong SAR, China; 2 The Centre for Research into Circulating Fetal Nucleic Acids, Li Ka Shing Institute of Health Sciences, The Chinese University of Hong Kong, Shatin, New Territories, Hong Kong SAR, China; 3 Department of Chemical Pathology, The Chinese University of Hong Kong, Shatin, New Territories, Hong Kong SAR, China; VU University Medical Center, Netherlands

## Abstract

**Background:**

Spontaneous preterm birth (SPB, before 37 gestational weeks) is a major cause of perinatal mortality and morbidity, but its pathogenesis remains unclear. Studies on SPB have been hampered by the limited availability of markers for SPB in predelivery clinical samples that can be easily compared with gestational age-matched normal controls. We hypothesize that SPB involves aberrant placental RNA expression, and that such RNA transcripts can be detected in predelivery maternal plasma samples, which can be compared with gestational age-matched controls.

**Principal Findings:**

Using gene expression microarray to profile essentially all human genes, we observed that 426 probe signals were changed by >2.9-fold in the SPB placentas, compared with the spontaneous term birth (STB) placentas. Among the genes represented by those probes, we observed an over-representation of functions in RNA stabilization, extracellular matrix binding, and acute inflammatory response. Using RT-quantitative PCR, we observed differences in the RNA concentrations of certain genes only between the SPB and STB placentas, but not between the STB and term elective cesarean delivery placentas. Notably, 36 RNA transcripts were observed at placental microarray signals higher than a threshold, which indicated the possibility of their detection in maternal plasma. Among them, the *IL1RL1* mRNA was tested in plasma samples taken from 37 women. It was detected in 6 of 10 (60%) plasma samples collected during the presentation of preterm labor (≤32.9 weeks) in women eventually giving SPB, but was detected in only 1 of 27 (4%) samples collected during matched gestational weeks from women with no preterm labor (Fisher exact test, p = 0.00056).

**Conclusion:**

We have identified 36 SPB-associated RNA transcripts, which are possibly detectable in maternal plasma. We have illustrated that the *IL1RL1* mRNA was more frequently detected in predelivery maternal plasma samples collected from women resulting in SPB than the gestational-age matched controls.

## Introduction

In developed countries, reported rates of preterm birth or delivery before 37 completed weeks of gestation, are generally 5–9% [1]. Of all preterm births, around 65% are spontaneous preterm births (SPB; resulting from spontaneous preterm labor and preterm pre-labor rupture of membranes), while the remaining are indicated preterm births delivered for medical reasons [2].

Genetic studies have reported associations of certain polymorphisms in individual genes involved in infection and inflammation with preterm birth [3]–[13]. Recently, a genome-wide linkage analysis performed in the Finnish families with recurrent SPB has revealed *insulin-like growth factor 1 receptor* (*IGF1R*) as a SPB susceptibility gene [14]. Replication of these association and linkage studies in larger cohorts and across populations is pending.

To shed light on the mechanism of SPB and search for markers to predict SPB, investigators have performed proteomic studies to identify the proteins that are differentially present in bodily fluids of pregnant women eventually giving SPB [15], [16], or having intra-amniotic infection, which is commonly associated with SPB [17], [18]. A study on the reproducibility of certain maternal serum peptides to correctly classify SPB has been planned using an independent cohort [15].

To the best of our knowledge, transcriptomic studies directly comparing the genome-wide gene expression profiles between the SPB placentas and the spontaneous term birth (STB) placentas have not yet been published. Published placental gene expression studies were often confounded by the different modes of delivery or underlying obstetric complications besides SPB [19], [20]. Thus, the expression profile of gene associated with SPB was not highlighted in those studies. In this study, we have compared the gene expression profiles in the placentas delivered by spontaneous births only, i.e. SPB and STB, but not indicated preterm births nor pregnancies with other obstetric complications. We have profiled the RNA levels of essentially all human genes in the SPB and STB placentas, using a gene expression microarray platform.

Previously, we and others have observed that the placenta releases its nucleic acids in maternal plasma [21], [22]. Based on the systematically profiling of gene expression in the placenta, we have discovered a panel of unreported pregnancy-associated RNA transcripts and microRNAs in maternal plasma, [23], [24]. Thus, in this study, we have also tested if the SPB-associated RNA transcripts, as identified systematically in the placenta using microarray, are detectable in maternal plasma. This may result in novel markers for SPB in maternal plasma.

## Materials and Methods

### Objectives

To investigate if SPB involves any aberrant gene expression in the placenta, we profiled the RNA expression levels of all human genes by microarray in the placentas collected from SPB, and compared with the data from STB. To decipher if certain genes were associated in (a) the pathogenesis of SPB, and/or (b) the normal term spontaneous labor process, we compared the reverse-transcriptase quantitative polymerase chain reaction (RT-qPCR) data on the placentas from (a) SPB versus STB, and (b) STB and term elective cesarean (TCS) delivery, respectively. To investigate if the SPB-associated RNA that could be detected before SPB, we analyzed maternal plasma samples collected during the presentation of preterm labor from women eventually giving SPB.

### Recruitment of participants

This study was conducted according to the principles expressed in the Declaration of Helsinki. Ethics approval from the Joint Chinese University of Hong Kong-New Territories East Cluster Clinical Research Ethics Committee and written informed consent from each participant were obtained. Pregnant women who were attending the Department of Obstetrics and Gynaecology, Prince of Wales Hospital, Hong Kong, and who were presenting with preterm (before 34 weeks) labor (Group I), undergoing spontaneous term (on or after 37 weeks) labor (Group II), undergoing elective term (≥37 weeks) cesarean delivery (Group III), or having no sign of labor before term (<34 weeks) (Group IV) were recruited for this study. Gestational age was established based on menstrual date confirmed by sonographic examination prior to 20 weeks gestation.

For each participant, a maternal blood sample was obtained immediately upon recruitment, while the placenta was obtained immediately upon delivery. Pregnancy outcomes, including the mode of delivery and maturity were followed up. Indicated preterm births, threatened preterm labor (term birth), multiple pregnancies, pregnancies complicated by preeclampsia, intrauterine growth restriction, fetal chromosomal and structural abnormalities were excluded from this study. Characteristics of the participants in this study are shown in Tables 1 and 2.

**Table 1:** Characteristics of participants in the analysis of placental tissues.

	Spontaneous preterm births (SPB)	Spontaneous term births (STB)	Term elective cesarean deliveries (TCS)	P-value^a^
Number of participants (*n*)	10	10	10	-
Maternal age in years (median, IQR)	32 (29–33)	31 (27–32)	30 (25–35)	0.703
Nulliparous (*n*, %)	6 (60%)	4 (40%)	4 (40%)	0.585
Weeks at delivery (median, IQR)	31.2 (27.0–32.6)	39.1 (38.4–40.0)	38.5 (38.4–38.6)	<0.001^b^
Birthweight in grams (median, IQR)	1468 (1135–1595)	3120 (2990–3595)	3203 (3010–3730)	<0.001^b^
Rupture of membrane >24 h before delivery (*n*, %)	4 (40%)	2 (20%)	0 (0%)	0.082
Antepartum hemorrhage (*n*, %)	2 (10%)	1 (10%)	0 (0%)	0.329

a Kruskal-Wallis test for continuous variables. Chi-square test for nominal variables.

b Student-Newman-Keuls test, p<0.05: SPB<STB, SPB<TCS.

**Table 2:** Characteristics of participants in the analysis of maternal plasma samples.

	SPB	Term birth	P-value^a^
Number of participants (*n*)	10	27	-
Maternal age in years (median, IQR)	32 (28–33)	32 (27–34)	0.707
Nulliparous (*n*, %)	6 (60%)	16 (59%)	1.000
Weeks at blood-taking (median, IQR)	30.9 (27.0–32.1)	31.0 (27.8–32.3)	0.891
Weeks at delivery (median, IQR)	30.9 (27.0–32.1)	39.1 (38.3–40.3)	<0.001
Birthweight (median, IQR)	1468(1135–1580)	3240(2976–3392)	<0.001
Rupture of membrane >24 h before delivery (*n, %*)	4 (40%)	3 (11%)	1.000

a Mann-Whitney rank sum test for continuous variables. Fisher exact test for nominal variables.

SPB, spontaneous preterm birth.

### Sample collection and processing

For each case in Groups I, II and III, a piece of placental tissue (about 0.1 cm^3^) was biopsied immediately after delivery, rinsed in phosphate buffered saline and stabilize in RNA later (Ambion), as previously described [23]. Care was taken to minimize the contamination of any maternal decidual tissue, the fetal membranes, and blood clots in the biopsied placental tissue. For each participant in Group I, a maternal peripheral blood sample (12 mL) was collected into tubes containing EDTA during presentation of preterm labor (<34 weeks), before the administration of steroids or tocolytics, if any. For each participant in Group IV, the maternal peripheral blood sample (12 mL) was collected during the antenatal visit at <34 weeks.

We stored the unprocessed blood samples at 4°C upon collection and harvested plasma from the blood samples within 6 h [25]. Plasma was harvested from EDTA-blood by a reported double-centrifugation protocol [26]. To improve the yield of RNA, the processed plasma (1.6 mL) was then mixed with Trizol LS reagent (Invitrogen) (4.8 mL), according to a recently reported protocol [27]. All processed placental tissue and plasma samples were stored at −80°C until RNA extraction. DNase I (Invitrogen) treatment was performed to remove genomic DNA contamination. The quantity and quality of the RNA preparations from placental tissue were assessed by spectrophotometer and Bioanalyzer (Agilent). The details are described in File S1.

### Gene expression microarray analysis

The relative expression levels of essentially all human genes were systematically profiled using the GeneChip Human Genome U133 Plus 2.0 Array (Affymetrix). Placental tissue collected from Groups I and II was subjected to RNA extraction and expression profiling on microarray. The microarray data were then analyzed by the GeneSpring GX software version 10 (Agilent). The details are described in File S1.

### Reverse-transcriptase quantitative polymerase chain reaction (RT-qPCR)

The concentrations of mRNA transcripts coding for *interleukin 1 receptor-like 1* (*IL1RL1*), *vascular endothelial growth factor A* (*VEGFA*), *insulin-like growth factor 2* (*IGF2*), *glutathione peroxidase 3* (*GPX3*), *nidogen 1* (*NID1*), *actin, gamma 2, smooth muscle, enteric* (*ACTG2*), *transgelin* (*TAGLN*), and *glyceraldehyde-3-phosphate dehydrogenase* (*GAPDH*) in placental tissues and maternal plasma were quantified by RT-qPCR. To minimize the effect of any contaminating genomic DNA in the RNA preparations, the RT-qPCR assay for all mRNA targets, except for *ACTG2*, were designed to be intron-spanning. However, due to certain constrains of the mRNA sequence, the RT-qPCR assay for *ACTG2* mRNA did not span any intron. Details of each mRNA target, the sequence and exon locations of each primer and probes, reaction conditions and thermal profiles are available in File S1, Tables S1 and S2.

The amplification of mRNA target was monitored and analyzed by an ABI Prism 7900 Sequence Detection System (Applied Biosystems) and Sequence Detection Software version 2.1 (Applied Biosystems). For each assay, a calibration was prepared by amplifying serial dilutions of HPLC-purified synthetic DNA oligonucleotides (Sigma-Proligo) representing the targeted amplicon at known concentrations. The PCR efficiency, slope, y intercept, correlation coefficient, dynamic range, limit of detection (LOD) and Cq variation at LOD are tabulated in Table S3, according to the guidelines on the Minimum Information for Publication of Quantitative Real-Time PCR Experiments (MIQE) [28]. Absolute concentrations of mRNA targets were calculated as the number of copies per ng of total RNA in placenta, or as the number of copies per milliliter of plasma. Concentrations of mRNA targets in a sample were analyzed relative to the *GAPDH* mRNA in the sample unless otherwise stated.

### Statistical analysis

Statistical analyses were performed with SigmaStat 3.5 software (Systat Software) using non-parametric tests unless otherwise stated.

## Results

### Genome-wide gene expression profiling of the SPB placentas

To test the hypothesis that SPB involves aberrant gene expression in the placenta, we performed microarray analysis on five placental tissue samples collected from SPB (Group I, SPB placentas, delivered at 24.4 weeks–33.0 weeks) and compared with another five placental tissue samples from STB (Group II, STB placentas, delivered at 38.4 weeks–40.0 weeks). Each sample was interrogated by 54675 probesets, representing the transcripts of essentially all human genes. Over 47000 transcripts and their variants, including those transcribed from more than 38500 well characterized genes and UniGenes, were interrogated by these microarray probes. To reduce the technical variations in the experimental procedures in handling different samples, the microarray data were normalized using the Robust Multiarray Average (RMA) method [29]. We identified 1549 probe signals, or gene expression levels, that were significantly changed between the preterm and STB placentas (Mann-Whitney Rank Sum Test, adjusted for multiple testing by the Benjamini and Hochberg method [30], adjusted p<0.05). The full dataset of this microarray experiment has been deposited at the Gene Expression Omnibus of the National Centre of Biotechnology Information (http://ncbi.nlm.nih.gov/GEO, accession number GSE18850). The quality control data for the microarray experiment are shown in Table S4.

To monitor the technical variation associated with labeling and hybridization in the microarray experiment, identical amounts of artificial RNA controls were spiked to each RNA sample before the labeling step. None of these spiked artificial RNA controls displayed more than 2.9-fold differences between the term and SPB placentas. Thus, we focused our analysis on probe signals with >2.9-fold difference, which might be attributed to the biological variation, rather than technical variation, between the term and SPB placentas. The microarray data showed that 426 probe signals were changed (240 were increased and 186 were decreased) by >2.9-fold in the preterm (SPB) placentas, compared with the term (STB) placentas. The lists of the up-regulated genes (Tables 3 and S5) and down-regulated genes (Tables 4 and S6) in the SPB placentas are tabulated in the descending order of the fold-change values.

**Table 3:** RNA transcripts that were up-regulated in the SPB placentas, relative to the STB placentas, according to the microarray experiment (Mann-Whitney test, adjusted by the Benjamini and Hochberg method, adjusted p<0.05).

Probeset ID	GenBank accession number	Gene symbol^a^	Gene name^a^	Fold-change up-regulated^b^
234066_at^c^	AL117622	*IL1RL1*	*interleukin 1 receptor-like 1*	15.5
212092_at^c^	BE858180	*PEG10*	*paternally expressed 10*	12.2
207526_s_at^c^	NM_003856	*IL1RL1*	*interleukin 1 receptor-like 1*	12.0
211948_x_at	BG261071	*PRRC2C*	*proline-rich coiled-coil 2C*	11.7
242870_at	BE551073	*RIMKLB*	*ribosomal modification protein rimK-like family member B*	11.4
204864_s_at	NM_002184	*IL6ST*	*interleukin 6 signal transducer (gp130, oncostatin M receptor)*	10.6
211000_s_at	AB015706	*IL6ST*	*interleukin 6 signal transducer (gp130, oncostatin M receptor)*	10.1
208003_s_at	NM_006599	*NFAT5*	*nuclear factor of activated T-cells 5, tonicity-responsive*	9.89
214055_x_at	AW238632	*PRRC2C*	*proline-rich coiled-coil 2C*	9.39
242809_at^c^	AI188516	*IL1RL1*	*interleukin 1 receptor-like 1*	9.38
244774_at^c^	R81072	*PHACTR2*	*phosphatase and actin regulator 2*	8.67
215342_s_at	AB019490	*RABGAP1L*	*RAB GTPase activating protein 1-like*	8.56
208719_s_at	U59321	*DDX17*	*DEAD (Asp-Glu-Ala-Asp) box polypeptide 17*	8.50
233011_at	AU155094	*ANXA1*	*annexin A1*	7.74
216493_s_at	AL023775	*IGF2BP3*	*insulin-like growth factor 2 mRNA binding protein 3*	7.05
…	…	…	…	…
			[Continues on Table S5 b.]	

a The RNA transcript interrogated by each probeset is shown as the gene symbol and name approved by the HUGO Gene Nomenclature Committee at the European Bioinformatics Institute (http://www.genenames.org). Certain probesets interrogating the same gene may be listed as different GenBank accession numbers. This may reflect the different isoforms being interrogated. Other details on the probesets are freely accessible at http://www.netaffx.com.

b Only 15 transcripts with the greatest fold-change values are shown. The remaining 225 transcripts are shown in Table S5.

c Up-regulated transcripts with higher expression levels than the *PLAC4* mRNA in the placenta, according to the microarray data.

SPB, spontaneous preterm birth; STB, spontaneous term birth.

**Table 4:** RNA transcripts that were down-regulated in the SPB placentas, relative to the STB placentas, according to the microarray experiment (Mann-Whitney test, adjusted by the Benjamini and Hochberg method, adjusted p<0.05).

Probeset ID	GenBank accession number	Gene symbol^a^	Gene name^a^	Fold-change down-regulated^b^
228697_at	AW731710	*HINT3*	*histidine triad nucleotide binding protein 3*	12.9
1558406_a_at	AK000786	*EXPH5*	*exophilin 5*	8.02
216766_at	AK025152	*PRKCE*	*protein kinase C, epsilon*	7.39
218002_s_at	NM_004887	*CXCL14*	*chemokine (C-X-C motif) ligand 14*	6.88
215191_at	AW836210	*KDM2A*	*lysine (K)-specific demethylase 2A*	6.63
1557512_at	BM664532	*CLIP1*	*CAP-GLY domain containing linker protein 1*	5.90
213939_s_at	AI871641	*RUFY3*	*RUN and FYVE domain containing 3*	5.65
215599_at	X83300	—	transcribed locus	5.61
233727_at	AL157472	—	transcribed locus	5.60
205594_at	NM_014897	*ZNF652*	*zinc finger protein 652*	5.58
210701_at	D85939	*CFDP1*	*craniofacial development protein 1*	5.38
216813_at	AL512728	—	transcribed locus	5.34
1557293_at	CA418406	—	transcribed locus	5.33
231644_at	AW016812	—	transcribed locus	5.24
1561180_at	AK021807	*LRP11*	*low density lipoprotein receptor-associated protein 11*	5.04
…	…	…	…	…
			[Continues on Table S6 b.]	

a If a probeset interrogates a RNA transcript with no gene symbol (“—”) approved by the HUGO Gene Nomenclature Committee at the European Bioinformatics Institute (http://www.genenames.org), it is shown as a “transcribed locus”. Other details on the probesets are freely accessible at http://www.netaffx.com.

b Only 15 transcripts with the greatest fold-change values are shown. The remaining 171 transcripts are shown in Table S6.

SPB, spontaneous preterm birth; STB, spontaneous term birth.

### Over-representation of functions among the lists of aberrantly expressed genes in the SPB placentas

To test if any gene functions were over-represented, or enriched, among the lists of up- or down-regulated genes in the SPB placentas, we performed the gene ontology (GO) analysis. First, each gene was mapped to its GO terms, which described the gene functions by a set of controlled vocabulary constructed by the Gene Ontology Consortium (AmiGO version 1.7, release 15 Jan 2011) [31], [32]. Second, for each GO term, its fold of enrichment was calculated as the ratio between its frequency in a list and its frequency in a background comprising all genes interrogated by the microarray (File S1). To test if the over-representation was statistically significant, a modified Fisher's exact test was performed, using the tools in the website of the Database for Annotation, Visualization, and Integrated Discovery (DAVID) [33] [34]. An error rate of 5% was applied to control the family-wide false discovery rate (FDR). The gene ontology terms enriched in the lists of up- (Table 5 and S7) and down-regulated genes (Table 6 and S8) in the SPB placentas are tabulated.

**Table 5:** Gene ontology (GO) terms over-represented in the list of genes that were up-regulated in the SPB placentas, compared with the STB placentas.

GO term: term definition (GO category)	Genes	Number of genes involved with this GO term and up-regulated	Total number of genes up-regulated	Number of genes involved with this GO term and on micro-array	Total number of genes on micro-array	Fold of over-representation	P
**RNA stabilization**: Prevention of degradation of RNA molecules. (biological process)	*DHX9, HNRNPU, VEGFA, SYNCRIP*	4	148	15	14116	25.4	0.000459
**extracellular matrix binding**: Interacting selectively and non-covalently with a component of the extracellular matrix. (molecular function)	*THBS1, ADAM9, VEGFA, NID1*	4	157	27	15143	14.3	0.00262
**actin filament**: A filamentous structure formed of a two-stranded helical polymer of the protein actin and associated proteins. Actin filaments are a major component of the contractile apparatus of skeletal muscle and the microfilaments of the cytoskeleton of eukaryotic cells. The filaments, comprising polymerized globular actin molecules, appear as flexible structures with a diameter of 5–9 nm. They are organized into a variety of linear bundles, two-dimensional networks, and three dimensional gels. In the cytoskeleton they are most highly concentrated in the cortex of the cell just beneath the plasma membrane. (cellular compartment)	*PALLD, EZR, IQGAP1, CALD1, FERMT2*	5	160	41	15908	12.1	0.00073

Terms with the highest fold of over-representation from each category are shown. The remaining terms are shown in Table S7.

**Table 6:** Gene ontology (GO) terms over-represented in the list of genes that were down-regulated in the SPB placentas, compared with the STB placentas.

GO term: term definition (GO category)	Genes	Number of genes involved with this GO term and down-regulated	Total number of genes down-regulated	Number of genes involved with this GO term and on micro-array	Total number of genes on micro-array	Fold of over-representation	P
**Neuron projection morphogenesis**: The process in which the anatomical structures of a neuron projection are generated and organized. A neuron projection is any process extending from a neural cell, such as axons or dendrites. (biological process)	*EGFR, BCL2, DST, PTK2, NRP1, SIAH1, CXCR4, NRCAM*	8	110	213	14116	4.82	0.00131
**Protein serine/threonine kinase activity**: Catalysis of the reactions: ATP+protein serine = ADP+protein serine phosphate, and ATP+protein threonine = ADP+protein threonine phosphate. (molecular function)	*TTBK2, SIK2, EGFR, NEK9, SNRK, PLK3, ATM, PRKAG2, TRPM6, PRKCE, MKNK1, PDPK1*	12	118	430	15143	3.58	0.000496
**Golgi apparatus part**: Any constituent part of the Golgi apparatus, a compound membranous cytoplasmic organelle of eukaryotic cells, consisting of flattened, ribosome-free vesicles arranged in a more or less regular stack. (cellular compartment)	*ATXN2, CUX1, ST3GAL1, SLC35A3, B4GALT1, CSGALNACT1, EXT1, TGOLN2, ARF1*	9	117	294	15908	4.16	0.0014

Terms with the highest fold of over-representation from each category are shown. The remaining terms are shown in Table S8.

### Systematic identification of SPB-associated RNA transcripts in maternal plasma

**Figure 1:**
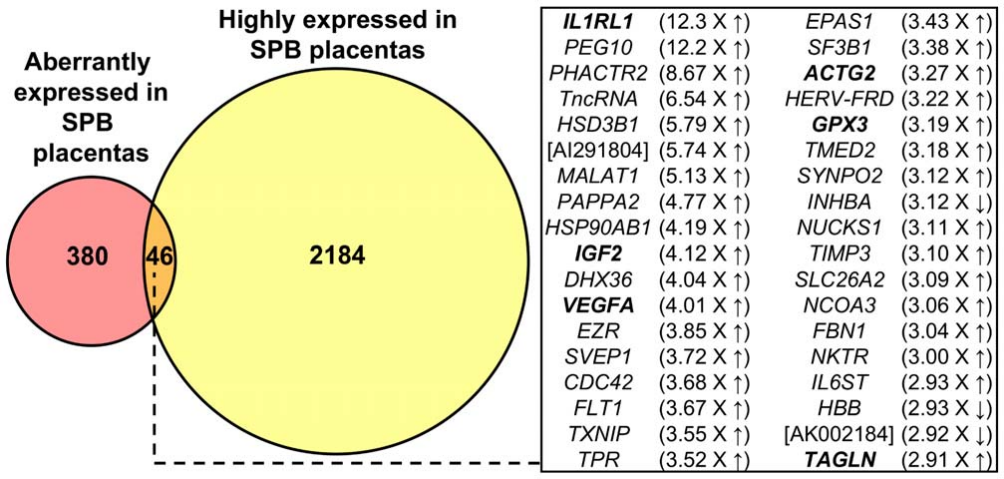
Systematic identification of SPB-associated placental transcripts in maternal plasma. Shown in the Venn diagram are the number of microarray probesets representing transcripts which are highly expressed (pale yellow) and aberrantly expressed (red) in the SPB placentas. Transcripts fulfilling both criteria (orange) are potentially detectable in maternal plasma samples obtained from women eventually resulting in SPB. These probesets represent 36 transcripts and are shown in the box, along with the fold-change up-regulated (up arrow) or down-regulated (down arrow) in the SPB placentas, compared with the STB placentas. For transcript represented by more than one probesets, its average fold-change is shown. Transcripts with no official gene symbols are shown in their GenBank accession numbers. Transcripts chosen for RT-qPCR are shown in bold. SPB, spontaneous preterm birth. STB, spontaneous term birth.

The data from our previous study suggest that placental expressed transcripts can be detected in maternal plasma provided that the expression level exceeds a threshold placental microarray signal [23]. The *PLAC4* mRNA was shown to be readily detectable in maternal plasma [35]. Among other placental mRNA that could be detected in maternal plasma, the *PLAC4* mRNA was expressed at a relatively low level in placental tissue and observed to yield a relatively low placental microarray signal [23]. Thus, in this study, we looked for transcript with higher expression levels than the *PLAC4* mRNA transcript in the placenta, because this indicated the possibility of detecting such transcripts in maternal plasma. In our current microarray data, the median microarray signal of the *PLAC4* mRNA in the STB placentas was 408 intensity units. We have identified 2230 microarray signals in the SPB placentas that were higher than this threshold, and referred them as “highly expressed” RNA transcripts (Figure 1, Table S9).

To systematically identify SPB-associated RNA transcripts in maternal plasma, we sought for transcripts that were both highly expressed in the SPB placentas and aberrantly expressed in the SPB placentas, compared with the STB placentas. Among these 2230 microarray signals representing the highly expressed placental RNA transcripts, 46 microarray signals (denoted in Tables 3, S5 and S6) were also found to be aberrantly expressed in the SPB placentas, compared with the STB placentas, and represented 36 placental RNA transcripts (Figure 1). Since this panel represents SPB-associated RNA transcripts that could potentially be detected in maternal plasma samples, we selected them for further investigation.

### Comparison of microarray data and RT-qPCR data

To assess if the fold-change values from this microarray dataset on a small sample size was reflective of the quantitative differences of expression levels in a larger sample size, we performed RT-qPCR on selected mRNA transcripts. Since most of the SPB-associated RNA transcripts that could potentially be detected in maternal plasma samples, as identified by the above method, were up-regulated, rather than down-regulated, in the SPB placentas, we focused on the up-regulated transcripts for further quantitative studies by RT-qPCR.

We selected 7 mRNA transcripts, namely those encoding *IL1RL1*, *VEGFA*, *IGF2*, *ACTG2*, *GPX3*, *NID1*, and *TAGLN* for RT-qPCR analysis in the RNA samples extracted from 10 placental tissues each collected from SPB (delivered at 24.4 weeks–33.1 weeks; Group I) and STB (delivered at 37.3 weeks–41.4 weeks; Group II) (Table 1). Among 10 placentas in each group, 5 of them were collected from a different set of women than those used in the microarray experiment. The 7 transcripts were selected because they covered a wide range of fold-change values in the microarray data (range, 2.90-fold to 15.5-fold). Moreover, all 7 transcripts, except the *NID1* mRNA, were identified as the SPB-associated placental RNA that could potentially be detected in maternal plasma (Figure 1).

As a control for the RNA extraction and equal RNA loading, we quantified the concentrations of *GAPDH* mRNA transcript in each group of the placental tissue RNA samples by RT-qPCR. No significant difference was observed in the concentrations of *GAPDH* mRNA between the two groups (Mann-Whitney rank sum test, p = 0.241). Subsequently, we compared the concentrations of the 7 selected transcripts, normalized to the concentrations of *GAPDH* mRNA, in this set of placental tissue RNA samples between Groups I and II.

The RT-qPCR data showed that the medians of normalized concentrations of *IL1RL1*, *VEGFA*, *IGF2*, *ACTG2*, *GPX3*, *NID1* and *TAGLN* were 8.96-fold (Mann-Whitney rank sum test, p = 0.014), 3.27-fold (p = 0.006), 1.55-fold (p = 0.076), 3.70-fold (p = 0.009), 2.31-fold (p = 0.002), 2.14-fold (p = 0.017) and 2.32-fold (p = 0.021) higher, respectively, in the placentas collected from SPB, than those collected from STB. All 7 mRNA transcripts, except *IGF2* mRNA, were significantly up-regulated in the SPB placenta relative to the STB placentas. For the 7 selected transcripts, their fold-change values according to the RT-qPCR data ranged from 2.32 to 8.96, which were comparable to those according to the microarray data (range 2.91 to 15.5, Figure S1). This microarray dataset on a small sample size was essentially concordant with the RT-qPCR data on a larger sample size.

### Further characterization of aberrantly expressed RNA in the SPB placentas

Genes that were up- or down-regulated in the SPB placenta as identified above may be associated specifically to the pathogenesis of SPB (preterm-associated), or to the normal physiology of a labor process (labor-associated), or a combination of both. To further characterize whether the 7 selected mRNA transcripts were preterm-associated and/or labor-associated, we quantified their mRNA expression levels in an additional group of placenta, namely those collected from elective TCS (delivered at 38.0–39.6 weeks; Group III), before the pregnant women underwent spontaneous labor, by RT-qPCR. The characteristics of participants in Group III are comparable to those of Groups I (SPB) and II (STB), except the gestational weeks at delivery, birthweights, and modes of delivery (Table 1). Since no systematic change was observed in the concentrations of *GAPDH* mRNA in the placentas from Groups I, II and III, we normalized the concentrations of the selected mRNA transcripts with the concentrations of *GAPDH* mRNA, and compare the data from the SPB, STB and TCS placentas.

**Figure 2:**
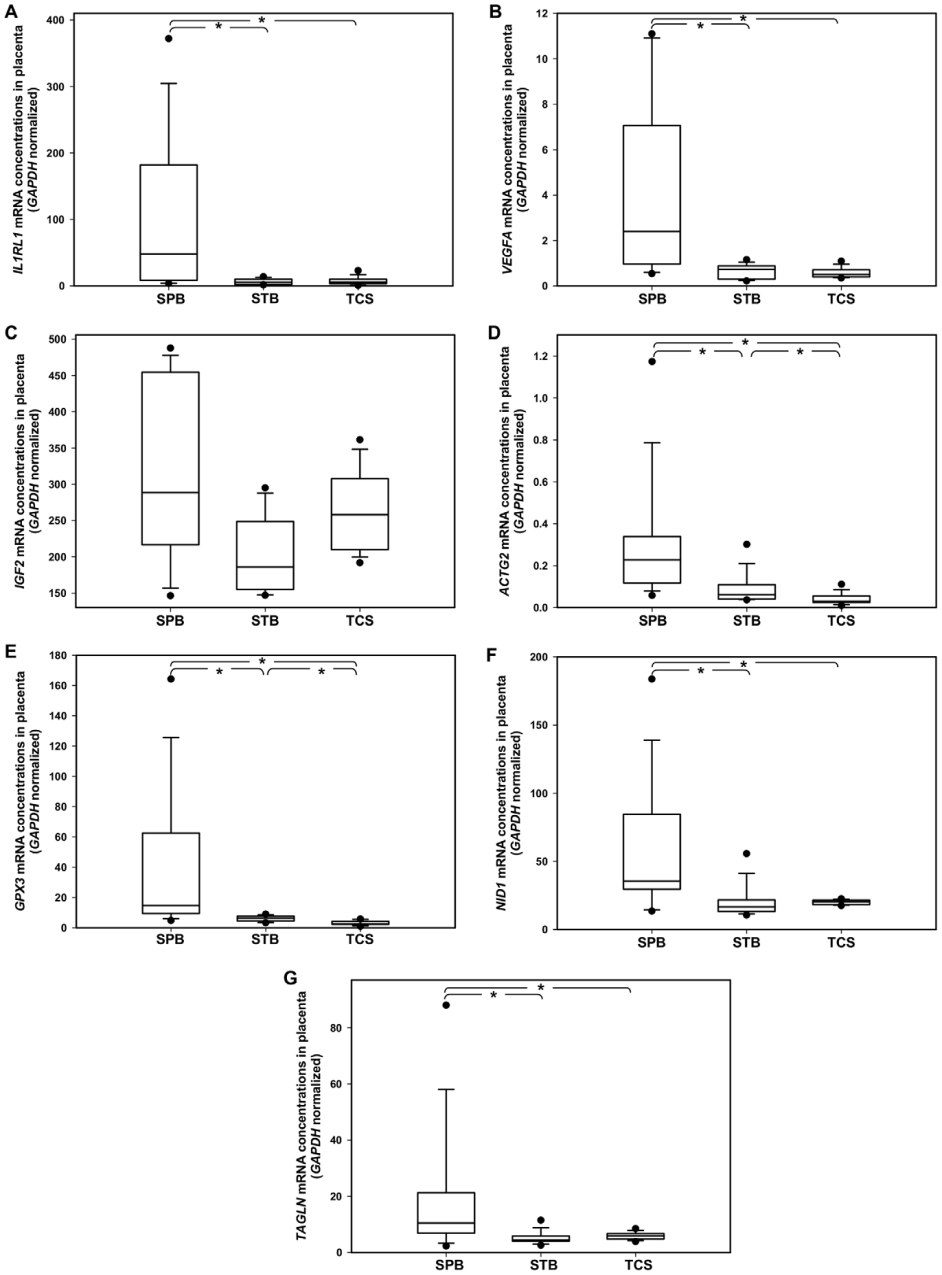
Box plots of the concentrations of RNA transcripts in the placentas as determined by RT-qPCR. Seven transcripts (A–F) with different fold-change values on the microarray dataset were selected for further investigation by RT-qPCR in the placentas collected from SPB, STB and TCS. The line inside each box denotes the median. Limits of the box denote the 25^th^ and 75^th^ percentiles. Whiskers denote the 10^th^ and 90^th^ percentiles. Filled circles denote the 5^th^ and 95^th^ percentiles. Asterisks denote the any pairwise comparisons that are significantly different (p<0.05, the Student-Newman-Keuls test). SPB, spontaneous preterm birth. STB, spontaneous term birth. TCS, elective term cesarean delivery.

To detect if there was any difference in the normalized mRNA concentrations among the three groups, the Kruskal-Wallis test was performed. Our data have shown that the normalized concentrations of all 7 selected mRNA transcripts, except the *IGF2* mRNA, were different among these three groups. To isolate any pairwise comparison with different normalized mRNA concentrations, the Student-Newman-Keuls test was performed. The normalized concentrations of all seven mRNA transcripts, except *IGF2* mRNA, were higher in the SPB placentas than the STB placentas (p<0.05, Student-Newman-Keuls; Figure 2). No difference was observed in the normalized concentrations of the *IL1RL1*, *VEGFA*, *NID1* and *TAGLN* mRNA transcripts between the STB placentas and TCS placentas (p>0.05, Student-Newman-Keuls; Figure 2A, B, F, and G). Only the normalized concentrations of the *ACTG2* and *GPX3* mRNA transcripts were higher in STB placentas, compared with the TCS placentas (p<0.05, Student-Newman-Keuls; Figure 2D, and E). Our data suggested that the *IL1RL1*, *VEGFA*, *NID1* and *TAGLN* mRNA transcripts are likely to be involved only in the pathogenesis of SPB (preterm-associated), but not the normal physiology of term labor (labor-associated).

### Detection of SPB-associated RNA transcripts in maternal plasma

Prompted by the results that aberrantly expressed RNA transcripts do exist in the SPB placentas, we further hypothesize that these RNA transcripts can be detected in maternal plasma before the occurrence of SPB. We obtained maternal plasma samples during preterm labor from women who eventually resulted in SPB (Group I) and during the same gestational weeks of blood-sampling from women with no preterm labor and who eventually resulted in term birth (Group IV).

We quantified the concentrations of *IL1RL1* mRNA, which showed the greatest fold-change in the SPB placentas compared with the STB placentas, in these maternal plasma samples using RT-qPCR. The LOD and the mean ± standard deviation of Cq for twenty technical replicates at LOD of our RT-qPCR assay for *IL1RL1* mRNA were 20 copies/mL plasma and 38.1±0.687 cycles, respectively. *IL1RL1* mRNA was considered to be detected only if the RT-qPCR signal was higher than the LOD, which was determined as the lowest concentration at which 95% of the positive samples are detected, in compliance with the MIQE guidelines [28].

Our data have shown that *IL1RL1* mRNA was indeed detectable in 6 out of 10 (60%) predelivery maternal plasma samples collected during preterm labor (blood collected at a median of 30.9 gestational weeks; IQR, 27.0 weeks–32.1 weeks) from women who eventually gave SPB (delivered at ≤33.1 gestational weeks), as we have hypothesized.

To test if the existence of *IL1RL1* mRNA in maternal plasma is specific to symptomatic women eventually resulting SPB, we collected gestational age-matched maternal plasma samples from 27 pregnant women with no labor and eventually resulted in term birth for comparison (blood collected at a median of 31.0 weeks; IQR, 27.8 weeks–32.3 weeks). The maternal and pregnancy characteristics of these 27 gestational age-matched pregnancies eventually resulted in term birth (delivered at <41.1 weeks) are comparable to the 10 women eventually resulted in SPB, except the gestational weeks at delivery and birthweights (Table 2).

Our data showed that *IL1RL1* mRNA was detected in only one of these 27 (3.7%) plasma samples of gestation in women with no labor eventually resulted in term birth. The detection rate of *IL1RL1* mRNA in predelivery maternal plasma samples was over 16 times higher in women with preterm labor and eventually resulted in SPB than those with no preterm labor and eventually resulted in term birth (Fisher exact test, p = 0.00056; Table 7). As a positive control for RNA extraction from each maternal plasma sample, we quantified the *GAPDH* mRNA and observed no difference in its plasma concentrations between the groups eventually resulted in SPB and term birth.

**Table 7:** Detection of *IL1RL1* mRNA in gestational age-matched maternal plasma samples.

*IL1RL1* mRNA	Number of women resulted in SPB^a^	Number of women resulted in term birth^b^
Positive (≥20 copies/mL maternal plasma)	6	1
Negative (<20 copies/mL maternal plasma)	4	26
Total	10	27

a Blood sample collected from women at 30.9 weeks (median; IQR, 27.0–32.1 weeks) of gestation, during the presentation of preterm labor (Table 2).

b Blood sample collected from women at 31.0 weeks (median; IQR, 27.8–32.3 weeks) of gestation (Table 2).

SPB, spontaneous preterm birth.

## Discussion

In this study, we have profiled the RNA expression levels of essentially all human genes in the SPB placenta, and observed that 426 microarray probe signals were changed in the SPB placentas, compared with the STB placentas (240 increased (Tables 3 and S5) and 186 decreased (Tables 4 and S6); Mann-Whitney test, adjusted for multiple testing by the Benjamini and Hochberg method, adjusted p<0.05). To the best of our knowledge, this is the first report that compared the genome-wide gene expression profiles of between the SPB and STB placentas. Notably, this dataset has also facilitated the first systematic identification of a panel of 36 SPB-associated RNA transcripts, which are potentially detectable in maternal plasma (Figure 1).

None of the previous transcriptomic studies compared the genome-wide profiles between the SPB and STB placentas, although one recent study compared the profiles between the SPB and the TCS placentas [19], which were collected from spontaneous delivery and elective cesarean delivery, respectively. Hence, the subsequent gene expression data might be confounded by the different modes of delivery. In contrast, our study compared the placental microarray data between SPB and STB, both of which had undergone spontaneous delivery. Thus, the differential gene expression patterns identified in our dataset are not confounded by the mode of delivery. Among the 426 SPB-associated genes identified in our study comparing SPB and STB placentas, only 5 were identified in the study comparing SPB and TCS placentas [19].

Besides the mode of delivery, previous microarray studies concerning SPB were often confounded by the underlying obstetric complications indicating for a preterm elective cesarean delivery. For instance, a recent microarray study compared the gene expression data between SPB and indicated preterm births by cesarean section at 32–34 weeks of gestation (indications: fetal growth restriction, placental abruption, placenta previa, spinal fusion) [20]. In contrast, we have excluded indicated preterm births from our study. Thus, the differential gene expression patterns identified in our dataset are neither confounded by the underlying obstetric complications in indicated preterm births.

To verify if our microarray experiment and our data-ming methods were performed properly, we repeated the measurement of gene expression levels using another platform, namely RT-qPCR, in a different set of samples. Seven transcripts with various fold-change values (range, 2.90-fold to 15.5-fold) on the microarray datasets were selected for further investigation by RT-qPCR in 20 pregnancies. The RT-qPCR data from 6 of 7 (86%) transcripts were concordant with the microarray data (Figure 2A, B, D–G; SPB *vs.* STB, p<0.05). The only discordant data on the *IGF2* mRNA (Figure 2C; SPB *vs.* STB, p≥0.05) may arise due to its multiple transcript variants, which require multiple variant-specific RT-qPCR assays for more accurate quantification. Nevertheless, this suggested that our microarray data on a small sample size is representative of the quantitative difference in RT-qPCR data in a larger cohort.

Furthermore, we have shown that the differential mRNA expression of gene as small as 2.9-fold on the microarray (*TAGLN*) could be replicated on the RT-qPCR platform in the larger cohort (Figure 2G; SPB vs. STB, p<0.05). Thus, we reason that the lists of up- and down-regulated genes (threshold, >2.9-fold on microarray; Tables 3, 4, S5, S6), identified by our microarray experiment and our data-mining methods, represent genes that are differentially expressed in the clinical samples.

Based on this SPB placental microarray dataset that is representative, not confounded by mode of delivery nor other obstetric complications, we further investigated into the mechanistic aspects of SPB in humans. Among the 7 transcripts tested by RT-qPCR, 4 transcripts (the *IL1RL1*, *VEGFA*, *NID1* and *TAGLN* mRNA) showed difference in concentrations only between the SPB placentas and the STB placentas, but no difference between the STB placentas and the TCS placentas (Figure 2A, B, F, and G; SPB *vs.* STB, p<0.05; STB *vs.* TCS, p≥0.05). This implies these RNA transcripts are involved specifically in the pathological physiology of SPB, but not in the normal physiology of labor process.

In fact, although the 2 other tested transcripts (the *ACTG2* and *GPX3* mRNA) showed higher concentrations in the STB placentas than the TCS placentas (Figure 2D, and E; STB *vs.* TCS, p<0.05), their concentrations were in turn higher in the SPB placentas than the STB placentas (Figure 2D, and E; SPB *vs.* STB, p<0.05). This implies that, while the concentrations of *ACTG2* and *GPX3* mRNA are changed in the normal physiology of labor process, their concentrations are further changed in the pathological physiology of SPB. Taken together, 6 of the 7 (86%) RNA transcripts identified by the above approach are predominantly associated with the pathological physiology of SPB.

To gain further insights into the known functions of genes up-regulated in the SPB placentas, compared with the STB placentas, we performed the GO analysis the list of genes in Tables 3 and S5. Not surprisingly, we observed an over-representation of gene functions involving acute inflammatory response (Table S7). This is in line with the literature that intrauterine infection or inflammation is implicated in the pathological processes of SPB [36]. Interestingly, we also observed an over-representation of gene functions involving RNA stabilization (Table 5). Recently, a compound with ribonuclease inhibitor activity has been shown to limit the growth of important gram-positive bacterial pathogens and protected against *S. aureus* pathogenesis in an animal model of infection [37]. We speculate that the up-regulation of genes involved in RNA stabilization (e.g. ribonuclease inhibitor activity) may be a response to invading pathogens in the SPB placentas.

Moreover, among the genes up-regulated in the SPB placentas, we observed an over-representation of gene functions in extracellular matrix binding (Table 5), including the *thrombospondin 1* (*THBS1*) and the *vascular endothelial growth factor A* (*VEGFA*) mRNA. The *VEGFA* protein has been found to be elevated in the decidual cells of women with intra-amniotic infection, which is a major cause of SPB [38]. The *THBS1* protein has been found to be elevated in the cervicovaginal fluids of women undergoing SPB, compared with those undergoing term births [39]. The *THBS1* mRNA has also been shown to be elevated during term and preterm parturition in the myometrium of sheep [40] and the uterus of mice [41]. Furthermore, gene functions involving with actin filament (Table 5) were also over-represented among the genes up-regulated in the SPB placenta, including *caldesmon 1* (*CALD1*), which is implicated in the stretch-dependent myometrial activation in human myometrium [42]. Thus, the involvement of the aberrant RNA expression of these genes in the SPB placenta, as revealed in this study, echoes well with their aberrant expression in the myometrium and other tissue collected from SPB, as reported in the literature.

Interestingly, we have observed that the *insulin-like growth factor 2 mRNA binding protein 3* (*IGF2BP3*) mRNA and the *IGF1R* mRNA were up-regulated by 7.05- and 3.39-fold, respectively, in the SPB placentas, compared with the STB placentas (Tables 3 and S5). Recently, a linkage, haplotype sharing, and association analysis has provided the evidence for *IGF1R* as a SPB predisposition gene [14]. Previously, a 11-kDa proteolytic fragment of the *insulin-like growth factor binding protein 1* (*IGFBP1*) protein was reported to be up-regulated in the amniotic fluid and maternal serum of pregnant women with intra-amniotic infection [17]. Taken altogether, *IGF* and its associated molecues may play some roles in the pathogenesis of SPB. In our GO analysis, among the up-regulated genes in the SPB placentas, the frequency of *IGF2BP3* and other genes involved in posttranscriptional regulation were 4.52-fold overrepresented (Table S7). Moreover, among the up-regulated genes, the frequency of *IGF1R* and 9 other genes involved in anti-apoptosis were 4.63-fold overrepresented (Table S7). Thus, we speculate that posttranscriptional regulation and anti-apoptosis may also be involved in the pathogenesis of SPB.

Most importantly, we have systematically identified a panel of 36 RNA transcripts which are aberrantly expressed in the SPB placentas, and are expressed at a high enough level to be detectable in maternal plasma (Figure 1). We have demonstrated that the *IL1RL1* mRNA, as identified above, was detected in 60% of predelivery maternal plasma samples collected during preterm labor (24–33 weeks 32.9 weeks) from women eventually resulted in SPB. In contrast, the *IL1RL1* mRNA was detected in only 3.7% of maternal plasma samples collected during the same gestational period in women not in labor. The data suggest that the existence of *IL1RL1* mRNA in the predelivery maternal plasma is associated with preterm labor and/or SPB. This promising data warrant further investigation. First, how early does the *IL1RL1* mRNA appear in maternal blood before SPB? Second, will the *IL1RL1* mRNA in maternal plasma improve the sensitivity of the existing methods, namely fetal fibronectin from cervicovaginal secretion and transvaginal cervical length [43], [44], in predicting SPB? Third, is *IL1RL1* mRNA in maternal plasma involved in all clinical sub-groups of SPB (e.g. with or without preterm pre-labor rupture of membranes, cervical incompetence, antepartum hemorrhage)?

As the other 35 SPB-associated RNA transcripts (Figure 1) are also identified as aberrantly expressed in the SPB placentas by our microarray experiment, further investigation, which may be achieved through maternal plasma detection, into their roles in SPB are warranted. For example, it would be interesting to correlate the maternal plasma concentrations of these SPB-associated RNA with any clinical outcomes/parameters of the pregnancies presented with preterm labor. Besides, to further study the molecular functions of the above genes in pregnancy and SPB, one may overexpress or knockout the concerned genes in animal models. Nevertheless, the work presented here has provided the essential groundwork for such further attempts to study SPB. Before this work, the difference between the genome-wide expression profiles of the SPB and STB placentas had not been compared, and systematic identification of markers for SPB in maternal blood had not been possible. These issues were addressed at least partially by the work presented here.

Furthermore, we have illustrated with our data that SPB-associated RNA in maternal plasma could be detected before SPB eventually occurred (Table 7). In contrast, markers derived from the myometrium and fetal membranes are not readily measurable for evaluation during preterm labor, or until SPB eventually occurs. The analysis of SPB-associated placental RNA transcripts in predelivery maternal plasma samples facilitates the fairer comparison of pregnancies presenting with preterm labor and gestational age-matched pregnancies with no preterm labor. We reason that this fairer comparison with gestational age-matched controls will in turn lead to discovery of better and more powerful markers in predicting SPB.

By providing this panel of 36 SPB-associated RNA markers in maternal circulation, we hope to alleviate the current lack of markers for SPB in predelivery clinical samples that can be easily compared with gestational age-matched normal controls. This strategy for the systematic identification of novel blood-based markers may also be generalized to other pregnancy-associated complications, which involve aberrant gene expression in the placenta. In summary, the current study has not only brought insights into the molecular biology of the placenta delivered by SPB, but also provided a list of SPB-associated RNA markers in maternal plasma for studying and monitoring SPB.

## Supporting Information

Figure S1 Fold-change values according to the microarray and the RT-qPCR platform.
(XLS)

Table S1 Gene symbols, gene names, and accession numbers of mRNA targeted by RT-qPCR assays.
(DOC)

Table S2 Primer/probes sequences and other details of RT-qPCR assays.
(DOC)

Table S3 Calibration curves for RT-qPCR assays.
(DOC)

Table S4 Quality control data of RNA samples submitted for microarray analysis.
(DOC)

Table S5 RNA transcripts that were up-regulated in the SPB placentas, relative to the STB placentas, according to the microarray data (Mann-Whitney test, adjusted by the Benjamini and Hochberg method, adjusted p<0.05; continued from Table 3).
(XLS)

Table S6 RNA transcripts that were down-regulated in the SPB placentas, relative to the STB placentas, according to the microarray data (Mann-Whitney test, adjusted by the Benjamini and Hochberg method, adjusted p<0.05; continued from Table 4).
(XLS)

Table S7 Gene ontology (GO) terms enriched in the list of genes that were up-regulated in the SPB placentas, compared with the STB placentas.
(XLS)

Table S8 Gene ontology (GO) terms enriched in the list of genes that were down-regulated in the SPB placentas, compared with the STB placentas.
(XLS)

Table S9 Transcripts that were expressed at higher levels than the *PLAC4* mRNA in the SPB placenta.
(XLS)

File S1 Supplementary methods.
(DOC)
