# Supplementary material for: Systematic Identification of Spontaneous Preterm Birth-Associated RNA Transcripts in Maternal Plasma
Source: PLoS One. 2012 Apr 5;7(4):e34328. doi: 10.1371/journal.pone.0034328 (PMC3320630; doi:10.1371/journal.pone.0034328)
Supplement: Table S1 — Gene symbols, gene names, and accession numbers of mRNA targeted by RT-qPCR assays. (DOC) [file pone.0034328.s002.doc]

**Supplemental Table S1: Gene symbols, gene names, and accession numbers of mRNA targeted by RT-qPCR assays**

| **Gene Symbol** | **Gene name** | **Reference sequence accession number** | **mRNA size (bp)** |
| --- | --- | --- | --- |
| *ACTG2* | Actin, gamma 2, smooth muscle, enteric | NM_001615.3 | 1345 |
| *GPX3* | Glutathione peroxidase 3 (plasma) | NM_002084.3 | 1779 |
| *IGF2* | Insulin-like growth factor 2 (somatomedin A), transcript variant 1 | NM_000612.4 | 5182 |
|  | Insulin-like growth factor 2 (somatomedin A), transcript variant 2 | NM_001007139.4 | 5156 |
| *IL1LR1* | Interleukin 1 receptor-like 1,transcript variant 1 | NM_016232.4 | 2058 |
|  | Interleukin 1 receptor-like 1, transcript variant 2 | NM_003856.2 | 2542 |
| *NID1* | Nidogen 1 | NM_002508.2 | 5903 |
| *TAGLN* | Transgelin (TAGLN), transcript variant 1 | NM_001001522.1 | 1574 |
|  | Transgelin (TAGLN), transcript variant 2 | NM_003186.3 | 1177 |
| *VEGFA* | Vascular endothelial growth factor A, transcript variant 1 | NM_001025366.2 | 3677 |
|  | Vascular endothelial growth factor A, transcript variant 2 | NM_003376.5 | 3626 |
|  | Vascular endothelial growth factor A, transcript variant 3 | NM_001025367.2 | 3608 |
|  | Vascular endothelial growth factor A, transcript variant 4 | NM_001025368.2 | 3554 |
|  | Vascular endothelial growth factor A, transcript variant 5 | NM_001025369.2 | 3519 |
|  | Vascular endothelial growth factor A, transcript variant 6 | NM_001025370.2 | 3422 |
|  | Vascular endothelial growth factor A, transcript variant 7 | NM_001033756.2 | 3488 |
| *GAPDH* | Glyceraldehyde-3-phosphate dehydrogenase | NM_002046.3 | 1310 |
| *APOLD1* | Apolipoprotein L domain containing 1, transcript variant 2 | NM_030817.2 | 4660 |
| *CSH1(hPL)* | Chorionic somatomammotropin hormone 1 (placental lactogen) | NM_001317.3 | 879 |
