# Supplementary material for: Systematic Identification of Spontaneous Preterm Birth-Associated RNA Transcripts in Maternal Plasma
Source: PLoS One. 2012 Apr 5;7(4):e34328. doi: 10.1371/journal.pone.0034328 (PMC3320630; doi:10.1371/journal.pone.0034328)
Supplement: Table S2 — Primer/probes sequences and other details of RT-qPCR assays. (DOC) [file pone.0034328.s003.doc]

**Supplemental Table S2: Primer/probes sequences and other details of RT-qPCR assays**

| **Assay name** | **Primer/probes sequences (5’ to 3’)** | **Final conc. (nM)** | **Primer start in ref. sequence**  **(base no.)** | **Location of amplicon**  **(exon no.)** | **Amplicon**  **size (bp)** | **Annealing temp. (°C)** |  | |
| --- | --- | --- | --- | --- | --- | --- | --- | --- |
| *ACTG2* | F: TTGCTCTGGTATTCATGCC | 400 | F: 18 (Exon 1) | Exon 1 | 58 | 58 |  | |
|  | R: GAGGTTCTTCTCCCAGTGACT | 400 | R: 55 (Exon 1) |  |  |  |  | |
|  | P: 5'-(FAM)-AAGACACACCAGCCCT(MGBNFQ)-3' ^a^ | 100 |  |  |  |  |  | |
| *GPX3* | F: TCTCGCAGAGCCGGG | 600 | F: 273 (Exon1) | Exon 1/2 | 69 | 57 |  | |
|  | R: GTAAATGGTGCCACTTATGCC | 600 | R: 320 (Exon2) |  |  |  |  | |
|  | P: 5'-(FAM)-ACAAGAGAAGTCGAAGATG(MGBNFQ)-3' ^a^ | 200 |  |  |  |  |  | |
| *IGF2* | F: ACACCCTCCAGTTCGTCTGTG | 400 | Transcript variant 1: | Transcript variant 1: | 69 | 58 |  | |
|  | R: GACGGCTCACACGGCTT | 400 | F: 868 (Exon2); R: 920 (Exon3) | Exon 2/3 |  |  |  | |
|  | P: 5'-(FAM)-CTTCTACTTCAGCAGGCC(MGBNFQ)-3' ^a^ | 100 | Transcript variant 2: | Transcript variant 2: | |  | |  |
|  |  |  | F: 842 (Exon3); R: 894 (Exon4) | Exon 3/4 |  |  |  | |
| *IL1LR1* | F: TCATGTATTCCACAGCAGCAA | 400 | Transcript variant 1: | Transcript variant 1: | 67 | 56 |  | |
|  | R: ACAATTAAAGCCTCATTTTCCAG | 400 | F: 306 (Exon2); R: 350 (Exon3) | Exon 2/3 | |  | |  |
|  | P: 5'-(FAM)-AGTTTAGTAAACAATCATGGGGC(MGBNFQ)-3' ^a^ | 100 | Transcript variant 2: | Transcript variant 2: | |  | |  |
|  |  |  | F: 265 (Exon2); R: 309 (Exon3) | Exon 2/3 | |  | |  |
| *NID1* | F: ACGACAGATCCGACATCGA | 400 | F: 299 (Exon 1) | Exon 1/2 | 65 | 58 |  | |
|  | R: GGGTTCACTCGTAGCAATGAT | 400 | R: 343 (Exon 2) |  |  |  |  | |
|  | P: 5'-(FAM)-CAGTCTACGTCACCACAAA(MGBNFQ)-3' ^a^ | 100 |  |  |  |  |  | |
| *TAGLN* | F: CGCTTGGGCTTCCAGGTC | 600 | Transcript variant 1: | Transcript variant 1: | 69 | 57 |  | |
|  | R: CAGGGTACAGGCTGTTCACCA | 600 | F: 663 (Exon 2); R: 712 (Exon 3) | Exon 2/3 |  |  |  | |
|  | P: 5'-(FAM)-AATGGCGTGATTCTGAG(MGBNFQ)-3' ^a^ | 200 | Transcript variant 2: | Transcript variant 2: |  |  |  | |
|  |  |  | F: 266 (Exon 2); R: 315 (Exon 3) | Exon 2/3 |  |  |  | |
| *VEGFA* | F: TTGCTGCTCTACCTCCACCAT | 400 | For all transcript variants: | For all transcript variants: | 71 | 58 |  | |
|  | R: TGATTCTGCCCTCCTCCTTCT | 400 | F: 1078 (Exon 1) | Exon 1/2 |  |  |  | |
|  | P: 5'-(FAM)-CAAGTGGTCCCAGGCT(MGBNFQ)-3' ^a^ | 100 | R: 1128 (Exon 2) |  |  |  |  | |
| *GAPDH* | F: GAAGGTGAAGGTCGGAGT | 200 | F: 108 (Exon 2) | Exon 2/4 | 226 | 60 |  | |
|  | R: GAAGATGGTGATGGGATTTC | 200 | R: 314 (Exon 4) |  |  |  |  | |
|  | P: 5'-(FAM)CAAGCTTCCCGTTCTCAGCC(TAMRA)-3' ^b^ | 100 |  |  |  |  |  | |
| *APOLD1* | F: TGCAGTGCGGGAGGAA | 400 | F :543 (Exon2) | Exon 2 | 63 | 58 |  | |
|  | R: CAAAGAAGACGATGAAGTAGACAGA | 400 | R :581 (Exon2) |  |  |  |  | |
|  | P: 5'-(FAM)-CCATCGCCCTGTACAA(MGBNFQ)-3' a | 100 |  |  |  |  |  | |
| *CSH1(hPL)* | F: CATGACTCCCAGACCTCCTTC | 600 | F: 330 (Exon 3) | Exon 3/4 | 98 | 56 |  | |
|  | R: TGCGGAGCAGCTCTAGATTG | 600 | R: 407 (Exon 3/4) |  |  |  |  | |
|  | P: 5'-(FAM)TTCTGTTGCGTTTCCTCCATGTTGG(TAMRA)-3' b | 200 |  |  |  |  |  | |
| *IL1LR1-78* | F: GATGCACCAGCATTTTTGAA | 400 | - | Transcript variant 1:  Intron region | 69 | 58 |  | |
|  | R: GACACTCCTCCCATCCTGAA | 400 | - | Transcript variant 2:  Promoter region |  |  |  | |
|  | P: 5'-(FAM)-AAGTCATAGATTTGGCCACAAA(MGBNFQ)-3' a | 100 | - |  |  |  |  | |

F=forward primer; R=reverse primer; P=probe

^a^ Fluorescent probes containing a reporter (FAM, 6-carboxyfluorescein) at the 5' end and a minor groove–binding nonfluorescent quencher (MGBNFQ) at the 3' end.

^b^ Dual-labeled fluorescent probes containing a reporter (FAM, 6-carboxyfluorescein) at the 5’ end and a quencher (TAMRA, 6-carboxytetramethylrhodamine) at the 3’ end.
