# Supplementary material for: Systematic Identification of Spontaneous Preterm Birth-Associated RNA Transcripts in Maternal Plasma
Source: PLoS One. 2012 Apr 5;7(4):e34328. doi: 10.1371/journal.pone.0034328 (PMC3320630; doi:10.1371/journal.pone.0034328)
Supplement: Table S3 — Calibration curves for RT-qPCR assays. (DOC) [file pone.0034328.s004.doc]

**Supplemental Table S3: Calibration curves*^a^* for RT-qPCR assays**

| **Assay** | **Slope*^b^*** | **y-intercept*^b^*** | **R^2^** | **PCR-Efficiency*^c^*** | **LOD**  **(per reaction) *^d^*** | **C_q_ variation at**  **LOD (CV)*^d^*** | **Dynamic range*^e^*** |
| --- | --- | --- | --- | --- | --- | --- | --- |
| *ACTG2* | -3.42 | 38.06 | 0.998 | 0.961 | 12.5 copies | 1.82% | 16.35-36.78 |
| *GPX3* | -3.34 | 39.32 | 0.997 | 0.993 | 12.5 copies | 2.94% | 17.78-38.15 |
| *IGF2* | -3.37 | 37.50 | 0.998 | 0.980 | 125 copies | 0.71% | 15.87-32.51 |
| *IL1LR1* | -3.37 | 38.64 | 0.999 | 0.980 | 4 copies | 2.72% | 17.20-39.12 |
| *NID1* | -3.19 | 38.68 | 0.997 | 1.058 | 12.5 copies | 6.44% | 17.93-37.22 |
| *TAGLN* | -3.41 | 39.20 | 1.000 | 0.965 | 12.5 copies | 4.09% | 17.45-37.81 |
| *VEGFA* | -3.36 | 38.13 | 0.999 | 0.984 | 12.5 copies | 2.59% | 16.51-36.61 |
| *GAPDH* | -3.63 | 34.27 | 0.997 | 0.886 | 1.9 pg | 1.04% | 21.02-35.06 |
| *APOLD1* | -3.49 | 37.81 | 0.998 | 0.934 | 12.5 copies | 1.11% | 15.34-35.90 |
| *CSH1(hPL)* | -3.63 | 42.33 | 0.998 | 0.886 | 75 copies | 1.24% | 19.24-37.18 |
| *IL1LR1-78* | -3.40 | 37.85 | 0.999 | 0.968 | 4 copies | 1.80% | 16.50-38.15 |

*^a^* The calibration curves for all mRNA targets, except *GAPDH*, were prepared by serial dilution of HPLC-purified single-stranded DNA oligonucleotides (Sigma-Proligos) corresponding to the mRNA sequence from 1.25×10^7^ copies/reaction to the stated copies/reaction well at the limit of detection (LOD). The calibration curve for *GAPDH* mRNA was prepared by serial dilutions of human control RNA (Applied Biosystems), with RNA concentrations ranging from 15000 to 1.9 pg/reaction.

*^b^* The slope, intercept, and R^2^ of the each calibration curve were calculated by the SDS 2.1 software (Applied Biosystems).

*^c^* The PCR-efficiency was calculated as (10^-1/slope^ - 1).

^d^ To determine the limit of detection (LOD), twenty replicated reactions were performed at the stated concentration and at least 95% of them showed positive signals. The coefficient of variation (CV) of the C_q_s of these 20 replicates was shown.

*^e^* Dynamic range represents the range of the C_q_ values between the highest and the lowest concentration of the generated calibration curve.
