# Supplementary material for: Systematic Identification of Spontaneous Preterm Birth-Associated RNA Transcripts in Maternal Plasma
Source: PLoS One. 2012 Apr 5;7(4):e34328. doi: 10.1371/journal.pone.0034328 (PMC3320630; doi:10.1371/journal.pone.0034328)
Supplement: Table S4 — Quality control data of RNA samples submitted for microarray analysis. (DOC) [file pone.0034328.s005.doc]

**Supplemental Table S4:** Quality control data of RNA samples submitted for microarray analysis.

| **Sample name** | **Description** | **Concentration (ng/μL)** | **Yield (ug)** | **A_260_/A_280_** |
| --- | --- | --- | --- | --- |
| SPB_Placenta_BioRep1 | Placenta, Preterm spontaneous delivery, Biological Replicate #1 | 1050 | 26.3 | 2.12 |
| SPB_Placenta_BioRep2 | Placenta, Preterm spontaneous delivery, Biological Replicate #2 | 739 | 18.5 | 2.09 |
| SPB_Placenta_BioRep3 | Placenta, Preterm spontaneous delivery, Biological Replicate #3 | 577 | 14.4 | 2.10 |
| SPB_Placenta_BioRep4 | Placenta, Preterm spontaneous delivery, Biological Replicate #4 | 608 | 15.2 | 2.05 |
| SPB_Placenta_BioRep5 | Placenta, Preterm spontaneous delivery, Biological Replicate #5 | 768 | 19.2 | 2.07 |
| STB_Placenta_BioRep1 | Placenta, Term spontaneous delivery, Biological Replicate #1 | 341 | 8.53 | 2.07 |
| STB_Placenta_BioRep2 | Placenta, Term spontaneous delivery, Biological Replicate #2 | 603 | 15.1 | 2.1 |
| STB_Placenta_BioRep3 | Placenta, Term spontaneous delivery, Biological Replicate #3 | 633 | 15.8 | 2.09 |
| STB_Placenta_BioRep4 | Placenta, Term spontaneous delivery, Biological Replicate #4 | 324 | 8.10 | 2.09 |
| STB_Placenta_BioRep5 | Placenta, Term spontaneous delivery, Biological Replicate #5 | 434 | 10.9 | 2.08 |
