# Supplementary material for: Systematic Identification of Spontaneous Preterm Birth-Associated RNA Transcripts in Maternal Plasma
Source: PLoS One. 2012 Apr 5;7(4):e34328. doi: 10.1371/journal.pone.0034328 (PMC3320630; doi:10.1371/journal.pone.0034328)
Supplement: File S1 — Supplementary methods. (DOC) [file pone.0034328.s011.doc]

**File S1. Supporting information (Chim *et al.*)**

**SUPPLEMENTARY METHODS**

**Sample collection and processing**

We centrifuged the EDTA-blood samples at 1,600 *g* for 10 min at 4^o^C, then transferred the plasma into separate plain polypropylene tubes without disturbing the underlying layer of blood cells, and further centrifuged the supernatant at 16,000 *g* for 10 min at 4^o^C (Centrifuge 5417R, Eppendorf) (Chiu et al., 2001). Every 1.6 mL of plasma was then mixed with 4.8 mL of Trizol LS reagent (Invitrogen) and stored at -80 ^o^C until extraction (Heung et al., 2009). Placental tissues stabilized in RNAlater solution (Ambion) promptly after collection were first stored at 4^o^C for 24 hours, and then at -80^o^C after aspirating out the RNAlater solution.

**RNA extraction**

For maternal plasma samples, each plasma-Trizol LS mixture (1.6 mL + 4.8 mL = 6.4 mL) was thawed, mixed with 1.28 mL of chloroform (Fisher Scientific, Leicestershire, UK; HPLC grade) and centrifuged at 12 000 *g* for 15 min at 4 °C. After phase separation, the aqueous layer was then carefully transferred to fresh polypropylene tubes. One volume of 700 mL/L ethanol (Merck KGaA, Darmstadt, Germany; Pro analysis grade, absolute) was added to one volume of the aqueous layer. The mixture was applied to a spin column of the RNeasy Mini Kit (Qiagen, Valencia, CA) and processed according to the manufacturer’s instructions. Total RNA was eluted in 50 µL of RNase-free water, and treated with Deoxyribonuclease I (Invitrogen; Amplification Grade) and stored at -80 °C until use.

For placental tissue, each sample was doubly extracted, first by Trizol reagent (Invitrogen), and then by RNeasy Mini Kit (Qiagen) as per the manufacturers’ instructions. After DNase I treatment, RNase inhibitor (RNasin, Promega) was added to minimize RNase degradation. RNA preparations from placental tissues were quality controlled by spectrophotometric readings at A_260_ and A_280_, measured in a buffer at neutral pH (NanoDrop, Thermo Scientific), and by the RNA 6000 Nano Chip kit on the 2100 bioanalyzer (Agilent). Key information including RNA concentration, yield, A_260_/A_280_, and electropherograms are summarized in Table S4 and Fig S1.

**Gene expression microarray analysis**

The RNA sample extracted from the placenta was further prepared and hybridized to the U133 Plus 2.0 Array according the Expression Analysis Technical Manual (Affymetrix). Specifically, the protocols on Eukaryotic Target Preparation (One-Cycle cDNA Synthesis with Spike-in Controls), and Eukaryotic Target Hybridization were followed. Each RNA sample was primed by oligo(dT) primer with T7 promoter sequence, reverse transcribed into cDNA, and subjected to *in vitro* transcription using the 3' IVT Express Kit (Affymetrix). The product was then hybridized, washed and stained using the GeneChip Hybridization, Wash, and Stain Kit (Affymetrix).

The data from each of the 5 preterm and 5 term placentas were normalized by the Robust Multiarray Average (RMA) method (Irizarry et al., 2003). The baseline was set to the median of each probeset. To filter out stochastic fluctuations from interfering the subsequent data analysis, we first performed a T-test (without multiple testing correction) on the probeset signals obtained in the preterm and term placentas. Among 54675 probesets, only 11587 were changed between the preterm and term placentas (T-test, p<0.05). Then, we further subjected these 11587 probesets to Mann-Whitney rank sum test with adjustment for multiple testing by the Benjamini and Hochberg method (Benjamini et al., 1995) (adjusted p<0.05).

Microarray probesets representing transcripts, which were changed by 2.9-fold or more were further analyzed by DAVID. If a probeset reprsented a transcript with no name and symbol ("---") approved by the HUGO Gene Nomenclature Committee at the European Bioinformatics Institute (http://www.genenames.org), it was listed as a "transcribed locus" only. The RNA transcript interrogated by each probeset is also listed as provided by the manufacturer (Affymetrix). Certain probesets interrogating the same gene might be listed as different GenBank accession numbers. This might reflect the different isoforms being interrogated. These details are freely accessible at http://www.netaffx.com.

**Analysis of gene ontology terms**

In each of the two gene lists (i.e. the lists of up- or down-regulated genes in the preterm placentas), every gene was first systematically mapped to its GO terms. Then, the frequency, *x*, of each GO term in the list was compared with the frequency, *y*, of that GO term in a background comprising all genes interrogated by the microarray. For a given GO term, *x* in the list was calculated as "the number of genes involved with the term and in the gene list" divided by "the total number of genes in the gene list". For that given GO term, *y* in the background was calculated as "the number of genes involved with the term and interrogated by the microarray" divided by "the total number of genes interrogated by the microarray". Also, the fold of enrichment of that given GO term was calculated by dividing frequency *x* by frequency *y*.

**Reverse-transcription quantitative polymerase chain reaction (RT-qPCR)**

**RT-qPCR was set up in a reaction volume of 25 µL using components supplied in an EZ rTth RNA PCR reagent set (Applied Biosystems). Different primer/probe concentrations were optimized for different assays (Table S1). Each reaction contained 5 µL of 5x EZ buffer, and final concentrations of 3 mM Mn(OAc)_2_, 300 µM each of dATP, dCTP, dGTP, 600 µM dUTP, 2.5 U of rTth polymerase, 0.25 U of *uracil N-glycosylase* (*UNG*). The final concentrations of primers and probes for each assay are stated in Table S2.**

**Extracted plasma RNA (5 µL) and 10 ng of extracted total blood cells/placental RNA were used for amplification. Each sample was analyzed in duplicate, and the corresponding calibration curve was run in parallel for each analysis.** No-template controls were performed in each reaction and no positive signal was observed. **Amplification data were collected and analyzed with an ABI Prism 7900 Sequence Detector (Applied Biosystems). Gene-specific primer and probe sequences are listed in Supplemental Table 2. All primers were supplied by** Integrated DNA Technologies.

**The thermal profile used for the RT-qPCR analysis was as follows: The reaction was initiated at 50°C for 2 min for the included *UNG* to act, followed by reverse transcription at 60°C for 30 min. After a 5-min denaturation at 95°C, 45 cycles of PCR were carried out by using denaturation at 94°C for 20 s and at the annealing/extension temperature stated for each assay (Table S2) for 1 min. Key information of the calibration curve for each assay is summarized in Table S3.**

**REFERENCES**

Benjamini, Y. and Hochberg, Y. (1995). "Controlling the false discovery rate: a practical and powerful approach to multiple testing." Journal of the Royal Statistical Society. Series B (Methodological) **57**(1): 289-300.

Chiu, R. W., Poon, L. L., Lau, T. K., Leung, T. N., Wong, E. M. and Lo, Y. M. (2001). "Effects of blood-processing protocols on fetal and total DNA quantification in maternal plasma." Clin Chem **47**(9): 1607-13.

Heung, M. M., Tsui, N. B., Leung, T. Y., Lau, T. K., Lo, Y. M. and Chiu, R. W. (2009). "Development of extraction protocols to improve the yield for fetal RNA in maternal plasma." Prenat Diagn **29**(3): 277-9.

Irizarry, R. A., Hobbs, B., Collin, F., Beazer-Barclay, Y. D., Antonellis, K. J., Scherf, U. and Speed, T. P. (2003). "Exploration, normalization, and summaries of high density oligonucleotide array probe level data." Biostatistics **4**(2): 249-64.
